# Stepping Towards Health: A Cross-Sectional Study of Hypertension, Mobility, and Endurance Among Saudi Adults Aged 50 Years and Older

**DOI:** 10.3390/jcm14238521

**Published:** 2025-12-01

**Authors:** Abdulfattah S. Alqahtani, Aqeel M. Alenazi

**Affiliations:** 1 Department of Rehabilitation Health Sciences, College of Applied Medical Sciences, King Saud University, Riyadh 12372, Saudi Arabia; 2 Department of Health and Rehabilitation Sciences, College of Applied Medical Sciences, Prince Sattam Bin Abdulaziz University, Al-Kharj 11942, Saudi Arabia; aqeel.alanazi@psau.edu.sa

**Keywords:** hypertension, physical function, older adults, functional performance, endurance, mobility

## Abstract

**Background/Objectives:** Hypertension (HTN) is highly prevalent among middle-aged and older adults in Saudi Arabia, affecting nearly half of those attending primary care clinics. This growing burden contributes not only to cardiovascular disease, but also to functional decline and reduced mobility in aging Saudis. The objective of this paper was to examine the relationship between HTN and objective measures of mobility and endurance in Saudi adults aged ≥50 years, and to assess whether any associations differ by sex. **Methods:** In a cross-sectional study, 47 hypertensive and 53 non-hypertensive (no chronic disease) community-dwelling adults were recruited from various regions of Saudi Arabia. Participants completed mobility tests (five repetitions of sit-to-stand (5×STS) and timed up-and-go (TUG)) and an endurance test (6 min walk test (6MWT)). Multivariable linear regressions adjusted for age, body mass index, and sociodemographic factors were used to evaluate the association of HTN with each performance measure in men and women separately. **Results:** Participants with HTN were older (mean 63 vs. 57 years) and had higher BMI than controls (*p* < 0.05), as well as performing worse on all functional tests: they required more time for 5×STS and TUG, and walked a shorter distance during the 6MWT (all *p* ≤ 0.003). In sex-stratified analyses, HTN was associated with slower TUG in men (≈2 s longer, *p* = 0.027), while among women, HTN predicted significantly slower 5×STS (+8.4 s) and TUG (+2.8 s) times, and a 114 m-shorter 6MWT distance (*p* < 0.05 each). **Conclusion:** HTN is linked to impaired mobility and endurance in middle-aged and older Saudi adults, with hypertensive women exhibiting particularly pronounced deficits. Future research is needed to determine whether incorporating routine functional assessments and exercise-based counseling into HTN care may help identify and address early declines in physical independence among aging adults.

## 1. Introduction

Hypertension (HTN) is a major public health challenge in aging populations. Globally, over 1.2 billion adults have HTN [1], and it is the leading preventable risk factor for cardiovascular disease and all-cause mortality [2]. The burden of HTN rises sharply with age: approximately half of adults ≥60 years are hypertensive [3]. In Saudi Arabia, HTN prevalence increased at different age groups, particularly among middle aged and older adults [4]. Life expectancy gains have led to a growing elderly population [3], and HTN is highly prevalent among older Saudis [4,5], affecting ~49% of those screened at primary care clinics [3]. Such high prevalence in late life is concerning not only due to cardiovascular complications, but also because HTN has been implicated in age-related decline in physical functions and symptoms alone, and with different coexisting conditions [6,7,8].

In the Saudi population, the development of hypertension is primarily linked to modifiable lifestyle and metabolic factors. Large national surveys have identified obesity, high dietary sodium intake, physical inactivity, dyslipidemia, and diabetes mellitus as the leading contributors to elevated blood pressure, while advancing age and family history remain prominent non-modifiable determinants [3,4,9,10]. Rapid urbanizations, increased consumption of processed foods, and sedentary behavior have intensified these risks, particularly among middle-aged adults. These characteristics highlight both shared and region-specific drivers of HTN compared with other global populations.

Maintaining mobility and endurance is critical for healthy aging and independence. Even modest impairments in gait speed or leg strength in older adults are associated with poorer quality of life, higher fall risk, and disability [6,11,12], and emerging evidence suggests that HTN may accelerate these functional declines. Longitudinal studies have shown that hypertensive older adults walk significantly more slowly than their normotensive peers and experience a faster age-related decline in gait speed [8,13]. The proposed mechanisms include HTN-induced vascular changes—such as arterial stiffness and cerebral small vessel disease—leading to reduced muscle perfusion, white matter lesions, and cognitive impairment, which in turn degrade mobility [11]. Indeed, hypertensive seniors tend to have more white matter lesions in the brain and poorer balance and cognition, correlating with slower timed up-and-go (TUG) performance and gait [14,15]. Beyond gait, HTN has also been linked to broader functional deficits; for instance, it is associated with higher odds of physical disability and falls in older adults [6].

Despite global research, the interplay between HTN and physical functions remains under-investigated in Middle Eastern populations. Sociocultural factors and lifestyle in Saudi Arabia (e.g., traditionally low physical activity, especially among women) [16,17,18] may modulate how HTN impacts functional ability. Furthermore, sex differences are important to consider. Men and women exhibit different profiles in both HTN and physical performance as they age. Older men have historically higher HTN prevalence until women reach menopause, after which women often catch up with and even surpass men in uncontrolled HTN rates [19]. With respect to physical function, healthy older males generally outperform females in tests of strength and endurance—for example, men walk longer distances in the 6 min walk test (6MWT) than women of the same age [20], largely due to greater muscle mass and stride length [21]. Women also tend to have higher body fat and lower muscle strength, which could exacerbate mobility limitations when chronic disease is present [22,23]. These disparities suggest that HTN might have differential impacts on mobility in men versus women. However, few studies have explicitly examined sex-specific associations between HTN and functional performance.

To address these gaps, the current study was conducted to evaluate the relationship between HTN and objective measures of mobility (five sit-to-stand repetitions [5×STS] and TUG) and an endurance test (6MWT) in community-dwelling Saudi adults aged 50 years and above. In Saudi Arabia, individuals within this age group often have the option of early retirement, a circumstance that may encourage sedentary behavior and consequently elevate their risk of developing chronic diseases such as diabetes, osteoarthritis, cardiovascular diseases, stroke, frailty, and reduced physical activity, particularly at relatively younger ages [9,10,18,24,25,26,27,28,29,30,31]. We also stratified analyses by sex to determine if hypertensive men and women experience similar or distinct functional deficits. Further, we hypothesized that participants with HTN would perform worse on mobility and endurance tests than those without HTN, and that this effect might be more pronounced in women, given their generally lower baseline physical ability. Understanding these patterns is clinically important: if HTN is linked with impaired mobility, comprehensive care for participants with HTN should include functional assessments and interventions to maintain and improve mobility. This study therefore seeks to shed light on the extent to which HTN contributes to mobility and endurance limitations in later life, within the context of an emerging older population in Saudi Arabia.

## 2. Materials and Methods

### 2.1. Design and Participants

**Figure 1:**
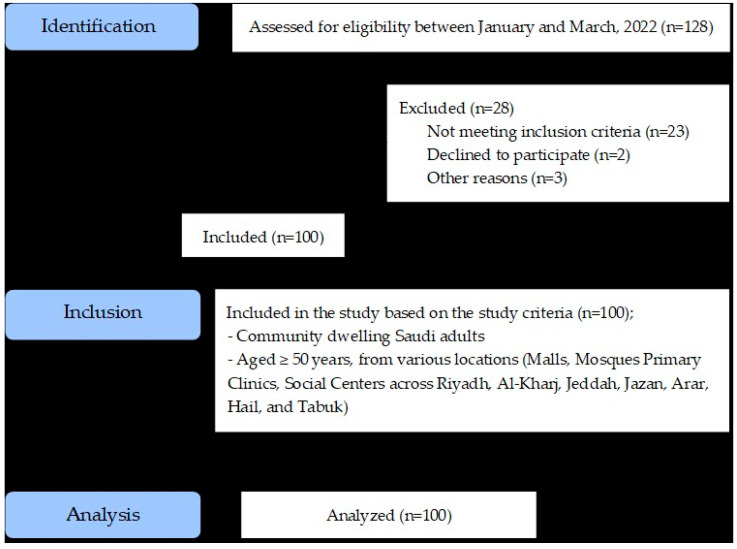
Participants flow diagram illustrates the screening, exclusion, and inclusion processes.

A non-probability convenience sampling method was employed to recruit community-dwelling Saudi adults aged ≥50 years from various public locations such as malls, mosques, primary clinics, and social centers across Riyadh, Al-Kharj, Jeddah, Jazan, Arar, Hail, and Tabuk between January 2022 and March 2022. Of the 128 individuals initially screened for eligibility, 28 were excluded; 23 out of 28 did not meet the inclusion criteria (inability to read and write Arabic and inability to perform the functional tests safely with or without assistive devices = 16 and age < 50 years = 7); 2 declined to participate; and 3 were excluded for other reasons, yielding a final analytic sample of 100 participants. A detailed participant flow diagram (Figure 1) illustrates the screening, exclusion, and inclusion processes.

For the purpose of the current study, which examined the association between HTN and mobility and endurance, only participants with self-reported HTN were included, and were then compared with a group of participants without HTN or any other chronic disease. Other participants were excluded from the analyses. Eligible participants received information regarding the study plan and objectives prior to signing the consent form.

To minimize selection bias, recruitment was conducted in multiple geographically distinct sites representing both urban and semi-urban populations. Measurement bias was reduced through uniform tester training, use of standardized test protocols, and double verification of data entry by two independent researchers. Analyses were adjusted for key confounders (age, BMI, education, marital status, employment) to control for potential covariate bias.

### 2.2. Ethical Consideration

The Research Ethics Committee at Prince Sattam bin Abdulaziz University has approved this study (No. RHPT/021/017), which was conducted in accordance with the Declaration of Helsinki (2010)’s ethical guidelines for conducting research and preserving the rights of humans involved in research. All participants were informed about the voluntary nature of participation in this study, with the option to withdraw at any point, and a signed informed consent form was obtained from each participant before it began.

### 2.3. Demographics and HTN

Demographical variables included age, sex, body mass index (BMI), marital status, education, and employment status. Age was recorded in years as a continuous variable and was verified by participants’ date of birth. Sex was dichotomized as male and female, while BMI was measured by dividing weight in kg by height square in meters. Education level was grouped into five categories: below elementary school, elementary school, middle school, high school, and university level or above. Finally, employment status was dichotomized as employed and non-employed/retired. HTN was obtained by self-report from the participants during the interview, including other chronic diseases such as arthritis, diabetes, cardiovascular diseases, dyslipidemia, anemia, osteoporosis, neurological conditions, and lower back pain.

### 2.4. Mobility Measures

#### 2.4.1. 5-Times Sit-to-Stand (5×STS)

This test is designed to measure lower-limb strength, mobility, and balance [32], and can be performed anywhere due to its usability, simple administration, and lack of requirements. A standard chair with back support was used with a standard height of approximately 40 cm, while a stopwatch was used to record the time required to perform the test. All participants were asked to perform the sit-to-stand task on the chair five times. A timer was started when the participant moved the back from the chair and stopped a standing position was reached on the fifth occasion. Lower time scores indicated better performance with high reliability and validity [32].

#### 2.4.2. Timed Up-And-Go (TUG)

This test is used to measure mobility, physical functions, and balance [33], and can be performed anywhere due to its simple administration. It requires a standard chair with armrests, a cone indicator for the 3 m distance, and a stopwatch to record time in seconds. Participants were instructed to rise from the chair, walk at their normal speed, turn around the cone, and walk back to sit on the chair. All participants completed three trials, with the first being a practice trial to familiarize them with the test without recording the time; the average of the two non-practice trials was used for the analysis. A lower time indicated better performance in the TUG test, which has high validity and reliability among community-dwelling older adults [33].

### 2.5. Endurance Measure

#### Six Min Walk Test (6MWT)

This test is used to measure aerobic performance and endurance: participants walk for a total of six minutes, and the distance is measured in meters [34]. All participants were allowed to rest while standing when needed, but the timer continued recording throughout, with the period and number of rests also being documented. Participants were allowed to use assistive devices or braces, and the choice to do so was documented for each participant. The turnaround points were marked by a cone, and participants were asked to wear comfortable clothes and shoes and to eat light meals before the test. While walking, participants were informed of every lapsed minute. A longer walking distance in meters indicated better performance, and the test has good reliability and validity across multiple populations [34].

To ensure data consistency across all collection sites, assessors were licensed physical therapists who received centralized training on standardized administration of the 5×STS, TUG, and 6MWT tests. All assessors used identical instructions, timing devices, and measurement tools, following a written protocol verified by the principal investigator before regional data collection commenced.

### 2.6. Sample Size Justification

The sample size was determined a priori using G*Power 3.1 for multiple linear regression, assuming a medium effect size (f^2^ = 0.15), α = 0.05, power = 0.80, and up to 5 predictors (age, BMI, marital status, education, and employment). The required sample was 92 participants, and considering possible attrition or unusable data (~10%), at least 100 participants were targeted and successfully analyzed, meeting the calculated requirement.

### 2.7. Statistical Analysis

Data were expressed as means with standard deviation (SD) for continuous variables or counts with percentages for categorical variables. Multiple linear regression analyses were conducted to examine the association between HTN, as a predictor variable, with mobility (5×STS and TUG) and endurance measures (6MWT), as dependent outcome variables for males and females, since sex was a significant factor for these outcomes. All analyses were adjusted for age, BMI, marital status, education, and employment status. Unstandardized coefficients (B) were created for each outcome variable, along with a 95% confidence interval. The alpha level was set at 0.05 for all analyses, and all 100 participants provided complete datasets for the three outcome measures; therefore, no cases were excluded from the final analyses. All analyses were conducted using IBM SPSS for Mac version 25.0 (SPSS Inc. Chicago, IL, USA).

## 3. Results

This study included a total of 100 participants with HTN (n = 47) and without HTN (n = 53). Table 1 compares the demographic data and clinical variables between those with and without HTN. In brief, participants with HTN had significantly higher age, BMI, and mobility measures, including 5×STS and TUG, compared to participants without HTN. More females were in the group with HTN compared to females without HTN.

Table 2 and Table 3 show the results of the multiple linear regression analyses examining the association between HTN with mobility and endurance measures for males and females, respectively, and HTN was only significant for one mobility measure (TUG) in males (B = 1.97, 95% CI [0.23, 3.70], *p* = 0.027). This indicates that male participants with HTN had an increase in TUG by 1.97 s after adjustments for age, BMI, marital status, employment status, and educational level. HTN was not associated with endurance measure (6MWT) in males, and female participants with HTN had significant increases in mobility measures, including 5×STS (B = 8.39, 95% CI [−2.82, 13.97], *p* = 0.003) and TUG (B = 2.76, 95% CI [0.20, 5.31], *p* = 0.034). Finally, females with HTN had a significant decrease in endurance measures, including 6MWT (B = −114, 95% CI [−193, −35], *p* = 0.005).

## 4. Discussion

In this cross-sectional study of Saudi adults aged 50 years and above, the findings indicated that HTN was associated with significantly poorer performance in standard mobility and endurance tests. The direction of findings was consistent across all functional measures—hypertensive individuals tended to take longer to rise from a chair five times (5×STS) and to complete the TUG, and they walked a shorter distance in 6 min compared to their normotensive counterparts—indicating overall lower physical function among those with HTN. However, when the results were stratified by sex and adjusted for covariates, an interesting pattern emerged: the deleterious impact of HTN on function was evident in both men and women, but was much more pronounced in women. Hypertensive women exhibited significantly slower chair rise and TUG times and a markedly reduced 6MWT distance relative to non-hypertensive women, whereas hypertensive men showed a significant deficit only in the TUG (and trend toward worse 5×STS and 6MWTs that did not reach the significance level). These results only partly supported our hypothesis, confirming that HTN is linked to mobility impairments in later life but suggesting that middle aged and older Saudi women with HTN bear a disproportionate burden of functional decline.

The current findings align with and expand on the prior literature on HTN and physical function. Several epidemiological studies have noted a connection between high blood pressure and reduced mobility in older adults [6,11]. For instance, Dumurgier et al. reported that hypertensive seniors in France’s Three-City study walked more slowly and had faster gait speed decline over 4 years than those with normal blood pressure [35]. Similarly, Rosano et al. found that higher blood pressure was associated with accelerated gait slowing over an 18-year period in well-functioning older adults [13]. Our cross-sectional results are consistent with these longitudinal observations: HTN corresponds to slower times in the TUG and shorter distances in the 6MWT. Notably, the TUG was significantly slower in hypertensive participants of both sexes in our cohort, reinforcing that HTN can impair even relatively simple mobility tasks. The TUG is known to reflect gait speed, balance, and overall mobility [33], and an extra 2–3 s (as observed in our hypertensive group) is clinically meaningful, potentially increasing fall risk [6]. The 6MWT distance was significantly lower in the HTN group only among women, but hypertensive men also showed a non-significant trend toward shorter distances. The lack of statistical significance in men’s 6MWT and 5×STS might be due to our modest sample size or the relatively high functional reserve in men blunting the observable impact of HTN. In women, however, HTN’s effect was clear and substantial: on average, hypertensive women walked ~114 m less and took ~8 s longer in the 5×STS test than normotensive women (adjusted results). These differences exceed typical test–retest variations and suggest true functional impairment.

The observed sex differences warrant discussion. Women in our study not only had a higher prevalence of HTN than men (consistent with national data on middle aged and older Saudis) [3,4], but those with HTN also experienced broader functional deficits. One explanation is that middle aged and older women generally have lower muscle strength and aerobic capacity than men, so the added burden of HTN (and possibly its treatment side effects) may push them past a threshold where function is noticeably reduced. It is well-documented that healthy adult females perform worse on physical performance tests compared to males of the same age [20,23]. For example, Steffen et al. found that community-dwelling women aged 70–79 walked on average ~56 m less in 6 min than men of that age [20], with this gap being largely attributed to men’s greater muscle mass, strength, and stride length [21]. In hypertensive individuals, these baseline sex disparities might be magnified. HTN can contribute to muscle perfusion deficits and inflammation that limit exercise capacity—effects made more evident by women’s lower reserve. Additionally, uncontrolled HTN and its comorbidities (like arterial stiffness) tend to rise in postmenopausal women [19], potentially leading to more pronounced end-organ damage affecting mobility (e.g., microvascular brain changes). Another consideration is physical activity levels: middle aged and older women in Saudi Arabia are known to be less physically active than men [16,17,18] due to cultural and lifestyle factors, which could exacerbate deconditioning in the presence of HTN. Indeed, physical inactivity is more common in females and older age groups in the region [16,18]. Thus, hypertensive women may enter older age with both a higher disease burden and lower fitness, a combination that manifests as significantly poorer endurance and strength performance. In contrast, middle aged and older men in our sample (who had greater muscle strength on average and a history of more active lifestyles) may have been better able to compensate for or mask the functional effects of HTN.

The mechanisms by which HTN might impair mobility and endurance are likely multifactorial. Chronic HTN leads to arterial stiffening and endothelial dysfunction, reducing blood flow to skeletal muscles during exercise and limiting oxygen delivery [36,37,38]. Over time, HTN also causes left ventricular hypertrophy and diminishes cardiac output reserve, which can curtail aerobic capacity (reflected in a shorter 6MWT) [39,40]. Hypertensive individuals commonly have subclinical cerebrovascular disease; for example, they accumulate white matter lesions in the brain at a faster rate [11,41]. These lesions are associated with gait and balance disturbances and slower processing speed, adversely affecting tests that require coordination like the TUG and 5×STS [11,41]. Our data cannot directly confirm mechanisms, but the robust association of HTN with slower TUG times in both sexes hints at a neural component (since TUG performance involves cognition and balance). Furthermore, many hypertensive patients take medications such as beta-blockers or diuretics that might contribute to fatigue, dizziness, or muscle weakness during physical tasks. It is possible that differences in medication use or HTN duration between the men and women in our study contributed to the sex-specific findings (though these details were not captured). Psychosocial factors may play a role as well—hypertensive adults might have anxiety about exertion or falls, leading to more cautious and slower performance on mobility tests. This could especially affect women if they have greater fear of falling [42].

Our results have practical implications for the care of middle aged and older adults with HTN. They underscore that hypertension is not only a leading cardiovascular risk factor but also a condition that can adversely affect mobility and endurance in middle-aged and older adults. Clinicians managing hypertensive patients in their 50s, 60s, and beyond should be encouraged to screen for functional decline. Simple tests like the 5×STS or TUG, which take only a few minutes, can be easily implemented in clinics to flag mobility problems. Notably, both tests are validated for middle aged and older adults [32,33], and poorer performance (e.g., TUG > 12 s) is known to correlate with higher fall risk and disability [6]. In hypertensive patients, detecting a decrement in these tests might prompt interventions such as referral to physical therapy or exercise programs. Our findings particularly suggest that hypertensive women should be targeted for early intervention to preserve function. For example, a 70-year-old woman with controlled HTN might still benefit from an individualized aerobic and resistance exercise regimen to improve her 6MWT distance and leg strength, thereby maintaining independence. Encouragingly, exercise has been shown to reduce blood pressure while simultaneously improving muscular fitness and walking endurance in older adults [5,43]. Thus, integrating lifestyle modification (physical activity promotion) into HTN management could yield dual benefits: better cardiovascular outcomes and better functional status. From a public health perspective, our results advocate for multidisciplinary care of hypertensive adults—involving geriatricians, cardiologists, and physiotherapists—to address both blood pressure control and mobility preservation. This may be especially relevant in countries like Saudi Arabia, where the aging population is rapidly growing [3] and rates of sedentary behavior are high [16,18].

Several strengths of this study enhance confidence in its findings. We specifically excluded individuals with comorbid chronic diseases (diabetes, arthritis, etc.) to isolate the association of HTN with physical function; this reduces confounding by other conditions that independently impair mobility. We also adjusted for key demographic and anthropometric factors (age, BMI, education, and marital and employment status), which accounted for baseline differences between the hypertensive and comparison groups. The use of objective, standardized tests (5×STS, TUG, and 6MWT) with proven reliability in middle aged and older populations lends credibility to our outcome measures [32,33,34]. Additionally, stratifying by sex provided novel insight into how gender may modify the HTN–function relationship.

Nonetheless, our study has limitations. The cross-sectional design precludes any causal inference; we cannot determine whether HTN led to poorer mobility or if individuals with worse mobility were simply more likely to report HTN (for instance, due to shared risk factors like obesity). Longitudinal studies are needed to confirm directionality, e.g., whether mid-life HTN predicts subsequent faster decline in walking ability. The sample size (n = 100, with subgroup analyses in 58 men and 42 women) was relatively small, which may have resulted in limited power to detect small effects, especially in men. Our participants were community-dwelling volunteers from various regions in Saudi Arabia, recruited from public places and clinics; while this broad recruitment enhances generalizability, it may also introduce selection bias (healthier or more health-conscious individuals might be more likely to participate). Importantly, hypertension was self-reported rather than clinically verified, which may introduce recall bias or misclassification errors. Such errors could attenuate the observed associations, meaning that the true relationship between hypertension and functional performance may be stronger than estimated. Some participants categorized as non-hypertensive may have undiagnosed or untreated high blood pressure, which would bias the results toward the null.

Additionally, we did not collect data on duration of HTN, medication type, or blood pressure control. These factors could influence physical performance (for example, a well-controlled hypertensive individual might perform better than someone with long-standing uncontrolled HTN), and physical activity level—a key determinant of both hypertension control and functional performance—was not assessed. Future studies should incorporate these clinical details to refine understanding, and should consider objective or self-reported physical activity measures to clarify whether reduced mobility among hypertensive individuals is mediated by lower habitual activity. Another limitation is the exclusion of those with other chronic illnesses to focus on the effect of HTN; while logical for internal validity, this limits external validity because in reality many middle aged and older hypertensive individuals have comorbidities (e.g., diabetes) that jointly affect mobility. Our findings, therefore, apply best to relatively healthier middle aged and older adults with HTN—the combined effect of multiple chronic conditions could be larger and merits investigation. Additionally, because individuals unable to safely complete the functional assessments were excluded, the observed differences between hypertensive and non-hypertensive participants may represent a conservative estimate of the true functional disparity within the broader older adult population.

Despite these limitations, our study contributes new knowledge by highlighting a clear link between HTN and reduced physical function in middle aged and older Saudi adults, with a differential impact by sex. To our knowledge, this is one of the first studies from the region to quantitatively demonstrate how a prevalent chronic disease (HTN) relates to functional mobility outcomes in the middle-aged and older population. The evidence that hypertensive women are particularly vulnerable to mobility loss is notable and should be explored further. Considering the sociocultural characteristics of the Saudi population, future interventions should explore the feasibility and impact of gender-specific programs that address the unique barriers and facilitators influencing participation among men and women. Designing and comparing the outcomes of such tailored exercise and lifestyle programs could provide more effective strategies for improving mobility, endurance, and overall health in hypertensive adults. It prompts questions for future research: Why are women more affected—is it biological (e.g., postmenopausal changes) or behavioral (lower activity, different care patterns)? Would aggressive blood pressure management or specific exercise interventions narrow the gap? Additionally, longitudinal research could examine whether treating HTN (and to what target) slows decline in gait speed or endurance, an area of interest given recent trials emphasizing intensive BP control in middle aged and older adults. There is also room to investigate the role of cognitive function in the HTN–mobility relationship; cognitive testing might reveal whether hypertensive individuals with executive function deficits perform disproportionately poorly in tasks like TUG that require cognitive input. Finally, expanding the sample to include those with comorbidities or frailty would provide insight into how HTN interacts with other factors to influence disability risk. Future studies should include objective verification of hypertension through clinical measurements and account for disease duration, medication types, and blood pressure control status to further enhance the robustness and clinical applicability of findings.

## 5. Conclusions

While this cross-sectional study highlights important associations between hypertension and reduced functional performance, particularly among women, causal relationships cannot be inferred. Hypertension in adults aged 50 years and above was associated with impaired mobility and endurance, as evidenced by slower sit-to-stand and timed-up-and-go performances, as well as shorter six-minute walking distances. Conducted among middle-aged and older Saudi adults, this study demonstrates that hypertensive individuals exhibit measurably poorer physical function than their normotensive peers, even after accounting for age and other factors. Notably, the adverse impact of hypertension on functional capacity appeared more pronounced in women, suggesting that middle-aged and older women with hypertension are at particular risk of mobility limitation.

These findings carry important clinical and public health implications. They emphasize the need for holistic management of hypertension in the aging population—beyond blood pressure control—to include regular monitoring of functional status. Simple interventions, such as incorporating strength and endurance training into hypertension care plans, may help mitigate the observed mobility deficits, particularly among female patients. In societies facing a rapidly growing older demographic, integrating cardiovascular and geriatric care strategies will be key.

Future longitudinal and interventional studies are warranted to determine whether targeted exercise programs and lifestyle modifications can reduce or reverse the mobility and endurance decline observed in hypertensive populations. Early screening and tailored physical activity programs may also inform national strategies promoting healthy aging and functional independence among Saudi adults.

## Figures and Tables

**Table 1:** Demographics and clinical characteristics (n = 100).

Variables	Hypertension (n = 47)	No Hypertension (n = 53)	*p*-Value
Age, years, mean ± SD	62.9 ± 9	57.1 ± 5	<0.001
Sex (female/male), count (%)	29 (61%)/18 (38%)	13 (24.5%)/40 (75.5%)	<0.001
BMI, kg/m^2^, mean ± SD	29.3 ± 5	27.3 ± 3	0.027
Marital status, count (%)			0.047
Married	38 (80.9%)	51 (96.2%)	
Divorced	3 (6.4%)	1 (1.9%)	
Widowed	6 (12.8%)	1 (1.9%)	
Educational level, count (%)			0.10
None	6 (12.8%)	1 (1.9%)	
Elementary school	7 (14.9%)	9 (17.0%)	
Middle school	8 (17.0%)	9 (17.0%)	
High school	16 (34.0%)	13 (24.5%)	
University level or above	10 (21.3%)	21 (39.6%)	
Employment status, count (%)			0.11
Employed	8 (17.0%)	17 (32.1%)	
Retired/unemployed	39 (83.0%)	36 (67.9%)	
5×STS, mean ± SD	16.1 ± 7	12.2 ± 3	0.001
TUG, mean ± SD	12.1 ± 3	9.6 ± 3	<0.001
6MWT, mean ± SD	390 ± 108	449 ± 78	0.003

Significant value if *p* ≤ 0.05; SD: standard deviation; BMI: body mass index; 5×STS: 5-times sit-to-stand; TUG: timed up-and-go test; 6MWT: 6 min walk test.

**Table 2:** Multiple linear regression for the association between hypertension and mobility and endurance measures for males (n = 58).

Variables	B (95% CI)	*p*-Value
5×STS	0.13 (−1.87 to 2.12)	0.90
TUG	1.97 (0.23 to 3.70)	0.027
6MWT	−1.46 (−46 to 44)	0.95

Significant value if *p* ≤ 0.05. All analyses were adjusted for age, BMI (body mass index), marital status, employment status, and educational level; B = unstandardized coefficient, CI = confidence interval, 5×STS = five-times sit-to-stand, TUG = timed up-and-go, 6MWT = six-minute walk test; all 95% confidence intervals and *p*-values were verified from the final regression outputs for internal consistency.

**Table 3:** Multiple linear regression for the association between hypertension with mobility and endurance measures for females (n = 42).

Variables	B (95% CI)	*p*-Value
5×STS	8.39 (2.82 to 13.97)	0.003
TUG	2.76 (0.20 to 5.31)	0.034
6MWT	−114 (−193 to −35)	0.005

Significant value if *p* ≤ 0.05. All analyses were adjusted for age, BMI (body mass index), marital status, employment status, and educational level; B = unstandardized coefficient, CI = confidence interval, 5×STS = five-times sit-to-stand, TUG = timed up-and-go, 6MWT = six-minute walk test; all 95% confidence intervals and *p*-values were verified from the final regression outputs for internal consistency.

## Data Availability

The original contributions presented in this study are included in the article. Further inquiries can be directed to the corresponding author.
